# Long-Term Histological Evaluation of a Novel Dermal Template in the Treatment of Pediatric Burns

**DOI:** 10.3390/bioengineering11121270

**Published:** 2024-12-14

**Authors:** Zeena Gerster-Barzanji, Vivienne Woodtli, Mira Klix, Thomas Biedermann, Clemens Schiestl, Kathrin Neuhaus, Melinda Farkas, Jivko Kamarachev, Daniel Rittirsch, Sophie Böttcher-Haberzeth

**Affiliations:** 1 Paediatric Burn Center, Children’s Skin Center, Department of Surgery, University Children’s Hospital Zurich, Lenggstrasse 30, 8008 Zurich, Switzerland; zeena.barzanji@uzh.ch (Z.G.-B.); vivienne.woodtli@kispi.uzh.ch (V.W.); mira.klix@kispi.uzh.ch (M.K.); clemens.schiestl@kispi.uzh.ch (C.S.); kathrin.neuhaus@kispi.uzh.ch (K.N.); melinda.farkas@kispi.uzh.ch (M.F.); 2 Children’s Research Center (CRC), University Children’s Hospital Zurich, University of Zurich, Lenggstrasse 30, 8008 Zurich, Switzerland; thomas.biedermann@kispi.uzh.ch; 3 Faculty of Medicine, University of Zurich (UZH), Rämistrasse 71, 8006 Zurich, Switzerland; jivko.kamarachev@usz.ch; 4 Tissue Biology Research Unit, Department of Surgery, University Children’s Hospital Zurich, Wagistrasse 12, 8952 Schlieren, Switzerland; 5 Department of Dermatology, University Hospital of Zurich, Wagistrasse 18, 8952 Schlieren, Switzerland; 6 Department of Plastic and Reconstructive Surgery, Klinik am Sonnenberg, Leibnizstrasse 19, 65191 Wiesbaden, Germany

**Keywords:** dermal template, NovoSorb^®^ Biodegradable Temporizing Matrix, burns, children, histological assessment, long-term analysis

## Abstract

For pediatric patients with full-thickness burns, achieving adequate dermal regeneration is essential to prevent inelastic scars that may hinder growth. Traditional autologous split-thickness skin grafts alone often fail to restore the dermal layer adequately. This study evaluates the long-term effect of using a NovoSorb^®^ Biodegradable Temporizing Matrix (BTM) as a dermal scaffold in four pediatric patients, promoting dermal formation before autografting. Pediatric burn patients treated at the University Children’s Hospital Zurich between 2020 and 2022 underwent a two-step treatment involving NovoSorb^®^ BTM application, followed by autografting. Histological analysis, conducted through 22 punch biopsies taken up to 2.6 years post-application, demonstrated robust dermal reorganization, with mature epidermal regeneration and stable dermo-epidermal connections. Immunofluorescence staining showed rapid capillary ingrowth, while extracellular matrix components, including collagen and elastic fibers, gradually aligned over time, mimicking normal skin structure. By 2.6 years, the dermal layer displayed characteristics close to uninjured skin, with remnants of NovoSorb^®^ BTM degrading within five months post-application. This study suggests that NovoSorb^®^ BTM facilitates elastic scar formation, offering significant benefits for pediatric patients by reducing functional limitations associated with inelastic scarring.

## 1. Introduction

The skin is a complex organ composed of the epidermis, dermis, and subcutis. The epidermis, originating from the ectoderm, consists of five stratified layers and connects to the mesoderm-derived dermis via the basement membrane and dermal papillae at the dermo-epidermal junction. While the dermis, composed predominantly of collagen and elastin, provides tensile strength and elasticity and accounts for 90% of skin thickness, the regeneration of this tissue presents significant challenges, primarily due to its dependence on scaffold structures in culture. These scaffolds are essential to facilitate fibroblast migration into the construct, enabling these cells to deposit collagen and establish the necessary extracellular matrix for functional tissue repair [1,2]. These challenges are particularly evident in the treatment of large, full-thickness wounds, where both the epidermal and dermal components are compromised. Such wounds demand advanced reconstructive strategies that go beyond traditional autologous split-thickness skin grafts (STSGs), which alone are insufficient to restore the skin’s functional, elastic, and aesthetic properties.

To overcome one challenge of limited donor areas, autografts with higher expansion rates are applied. Methods include meshed STSG, application of MEEK micrografts, or—if available—cultured autografts spanning from simple cultured epidermal or dermo-epidermal autografts (CEA/CDEA) to more sophisticated cultured full-thickness skin substitutes, such as denovoSkin™ (dS), a product currently being successfully tested in a phase II clinical trial [3,4]. As production of cultured autografts is time consuming, a bridging method, such as allografts or dermal templates, to cover the excised areas until cultured skin is available, is needed.

For another challenge, the reconstruction of the insufficient dermal component, a specific dermal regeneration is required, for which templates can be applied. These, firstly, serve as immediate coverage on excised areas, limiting the hypermetabolic condition of the severely burned patient and reducing risks of infection, and secondly, they facilitate the auto-regeneration of a dermis by providing a matrix as a template [5]. Until scientific development has reached the point that cultured full-thickness skin is available rapidly after the trauma and in large quantities, a two-staged method seems the most suitable option for the treatment of most large full-thickness skin defects. Dermal templates are used with increasing popularity in a two-step procedure [6]. With their impermeable seal providing immediate temporal wound closure and their dermal matrix promoting dermal regeneration, they render surgeons more time for definitive wound closure in a second planned step, for instance when donor sites that have been used for another area have recovered for reharvesting [5]. Among these, dermal substitutes such as the Integra^®^ Dermal Regeneration Template, introduced in the 1970s, have set a benchmark in full-thickness burn management by providing a bilayer structure comprising a collagen matrix and silicone layer for robust dermal regeneration. MatriDerm^®^, a single-layer bovine collagen and elastin scaffold, has shown promising results in promoting dermal regeneration, particularly in one-step procedures combined with STSG, though its susceptibility to infection and limited barrier properties remain challenges. Similarly, AlloDerm^TM^ RTM, a decellularized human dermis, facilitates dermal regeneration and integration with STSG but is limited by its cost, potential for disease transmission, and ethical concerns associated with human tissue sourcing [7]. In summary, biomaterial-based matrices are known to underlie a costly production process and only provide a limited barrier against pathogens, with reported infection rates as high as 16.9% [8].

NovoSorb^®^ Biodegradable Temporizing Matrix (BTM), a fully synthetic dermal substitute, addresses many limitations associated with biological and human-derived matrices. Composed of a biodegradable polyurethane foam with a non-biodegradable sealing membrane, BTM provides immediate wound coverage, promotes neodermis formation, and reduces infection risk due to its synthetic composition. Additionally, it is more cost-effective than natural biomaterials while delivering comparable functional and aesthetic outcomes [7,8,9,10]. Several studies have been conducted to demonstrate the efficacy of NovoSorb^®^ BTM in both preclinical and clinical settings [9,11,12,13,14,15,16,17,18,19]. However, the development of a “neodermis” following the gradual degradation of NovoSorb^®^ BTM is not fully understood yet. While most histological studies are confined to in vitro and animal models [5,20], clinical histological analyses after NovoSorb^®^ BTM application are, to our knowledge, only mentioned in a single case of an adult burn patient [21] as well as a pediatric patient [22].

This study is designed as an observational longitudinal study aimed at gaining a deeper understanding of neodermal development by evaluating the histological characteristics of skin biopsies from pediatric burn patients treated with NovoSorb^®^ Biodegradable Temporizing Matrix (BTM) and covered with various autografts. The evaluation is conducted at multiple time points, extending up to 2.6 years post-NovoSorb^®^ BTM application. This approach allows for temporal analysis of neodermal regeneration and autograft integration, contributing valuable insights into the long-term properties and interaction dynamics between NovoSorb^®^ BTM and autografts in a pediatric cohort.

## 2. Materials and Methods

### 2.1. Patients

Four pediatric patients with large full-thickness thermal injuries treated in a two-step procedure with NovoSorb^®^ BTM and subsequent coverage with an autograft at the University Children’s Hospital Zurich between January 2020 and September 2022 were included in this observational, longitudinal study and a total of 22 biopsies were taken. Patient demographics, characteristics, treatment sequence, and grafting modalities of the thermal injury were gathered using the information from medical records. Photographic documentation of the transplanted areas was performed routinely. Four mm punch biopsies of the transplanted areas were taken during routine procedures at various time points after application of NovoSorb^®^ BTM and prepared for histological assessment as described below. This study focused on pediatric patients, wherein biopsies were obtained exclusively during surgical procedures performed under general anesthesia. There was no standardized protocol guiding the timing or frequency of biopsy collection. Instead, biopsies were acquired opportunistically, coinciding with surgical interventions that were clinically indicated. Consequently, the availability of biopsy specimens was inherently dependent on the timing and necessity of these surgical procedures at specific locations and time points.

According to the regional Ethics Committee (Kantonale Ethikkommission Zurich, Switzerland; BASEC-Nr Req-2021-01431), the research project was not within the scope of the Human Research Act (HRA) and therefore needed no further approval. Parents, patients, and/or legal representatives gave written informed consent to all biopsies and pictures taken for this research project.

### 2.2. Material

NovoSorb^®^ BTM is a fully synthetic dermal template consisting of a biodegradable polyurethane foam layer and an outer non-biodegradable sealing membrane. It was approved by the FDA for the treatment of skin defects and surgical wounds in 2015 in the US, was granted CE mark approval for sale in Europe in 2019 [23], and for Switzerland in 2020 [24]. At the University Children’s Hospital, it is used—as intended—in a two-step procedure with placement on the excised defect in a first step. Dressing changes are performed weekly to ensure adherence and to check for its vascularization progress. After satisfying vascularization of NovoSorb^®^ BTM is achieved, approximately as of three to five weeks after application, the sealing membrane is removed, and the autograft is applied in a second step. CEAs or CDEAs are cell sheets produced from a small patient biopsy as described by Chemali et al. [25]. For patient biopsies analyzed here, CEAs and CDEAs were produced at the Centre de Production Cellulaire in Lausanne, Switzerland. After a production time of three to four weeks, CEAs or CDEAs are transferred to a vaseline gauze with which they are applied to the patient [26].

DenovoSkin™, also known as Zurich Skin, is a bioengineered new approach to skin grafts requiring a small biopsy of the patient’s healthy skin for fabrication. Thereby, keratinocytes and fibroblasts are isolated from the biopsy, cultured in vitro, and then assembled within a hydrogel for tissue formation as described in Schiestl et al. [3]. After a production time of 3–4 weeks, it is applied onto the prepared wound bed. A phase I clinical trial to demonstrate safety has been completed successfully [3,4]. Currently, phase II clinical trials are ongoing to compare dS to conventional split-thickness skin grafts [27].

### 2.3. Histological Assessment

Punch biopsies were prepared for immunohistochemistry and -fluorescence, as well as hemotoxylin and eosin (H&E) and Elastica van Gieson (EvG) staining to visualize different components of the epidermal and dermal layer. H&E staining is primarily used to assess the structural organization of skin treated with NovoSorb^®^ BTM, including the epidermis and “neodermis” or residual dermal layer. Immunofluorescence was applied to visualize vasculature, immune cells, and the dermo-epidermal junction, while Elastica van Gieson staining offered a more sophisticated understanding of elastic and collagen fiber distribution.

For immunofluorescence and H&E stainings, biopsies were embedded in Tissue-Tec O.C.T. Compound (Sakura Finetek, USA), cut into 12 μm sections (cryotome, Leica, Switzerland), and placed onto glass slides (Leica). Slides were stored at −80 °C and incubated for 30 min at 42 °C before histological staining. For EvG stainings biopsies were embedded in paraffin, cut in 2 μm sections, and placed onto SuperFrost slides. Slides were stored at −20 °C and incubated for 30 min at 42 °C before histological staining.

### 2.4. Hematoxylin and Eosin

Slides were pre-heated for 30 min at 42 °C and then dipped in hematoxylin (Sigma-Aldrich, Buchs, Switzerland) for 1 min. Afterwards, they were rinsed with H_2_O. Subsequently, slides were dipped about 10 s in eosin (Sigma-Aldrich) and afterwards rinsed with H_2_O. Next, slides were put in 50% ethanol for about 1 min and then in 70%, 80%, 95%, and finally 100% for 1 min each. Lastly, slides were put in xylol for 2–3 min. Afterwards, slides were covered with 3–4 drops of mounting medium and a cover slip was applied. Images were taken with a Nikon Eclipse Ti2 (Nikon, Switzerland) microscope.

### 2.5. Elastica Van Gieson

Firstly, slides were deparaffinized and, secondly, put in resorcin fuchsin solution for 30 min. Slides were put shortly in HCl-alcohol 0.1% and then washed with distilled water. Next, slides were put in Weigert’s iron hematoxylin staining solution for 2 min and then put in water for 5 min. Again, they were washed with distilled water. Thirdly, slides were put in picrofuchsin solution for 2 min. Slides were dried by paper towels and dehydrated in 100% alcohol twice, and lastly, slides were put in xylene twice for 5 min each. Slides were then covered with non-aqueous mounting agent and a cover slip.

### 2.6. Staining Protocol for Immunofluorescence

Slides were pre-heated for 30 minutes at 42 °C. Subsequently, skin sections were washed 3 times for 5 min each in PBS (Thermo Fisher, Buchs, Switzerland). Following this, the sections were fixed in methanol/aceton (1:1, Sigma Aldrich, Switzerland) for 5 min. Afterwards, blocking of the skin sections was performed for 30 min at room temperature (RT) with 2% BSA/PBS (Sigma-Aldrich, Basel, Switzerland). Next, the primary antibody was added and incubated at −20 °C overnight. The next day, slides were washed 3 times for 5 min each in PBS. Another blocking step by 2% BSA followed for 15 min. Subsequently, the secondary antibody was added for 1 h at RT. Afterwards, slides were washed 3 times for 5 min each in PBS and blocked with 2% BSA for 15 min. Finally, pre-labeled antibodies were added for 40 min at RT. The last steps included another washing step: 3 times for 5 min each. Finally, mounting medium containing DAPI (Sigma-Aldrich) was added, and slides were covered with a cover slip.

Images were taken with a DXM1200F digital camera connected to a Nikon Eclipse TE2000-U inverted microscope. The device is equipped with FITC- and TRITC-filter sets (Nikon AG, Egg, Switzerland; software: Nikon ACT-1 vers. 2.70) and the images taken were processed using ImageJ (version 2.3.0/1.53q).

#### Antibodies

The following primary antibodies were used for immunofluorescence staining: anti-Fibronectin (polyclonal, 1:50, Abcam), anti-Laminin 5 (clone: P3H9-2, 1:100, Santa Cruz). Anti-CD31 was pre-labeled for double immunofluorescence with AlexaFluor 488 (clone: WM59, 1:200, BD Pharmingen), as secondary antibody AlexaFluor 568 (polyclonal, 1:200, Abcam) was used.

### 2.7. CD68

The CD68 staining protocol utilizing the UltraView Red procedure on the BenchMark ULTRA IHC/ISH staining module begins with a baking step, where the slide is heated to 62 °C and incubated for 8 min. Following this, the deparaffinization function is activated, raising the slide temperature to 72 °C. The heat pretreatment then commences using the ULTRA Conditioner #1 program, heating the slide to 95 °C for an 8 min incubation. Cell conditioning follows, applying ULTRA CC1 for sequential periods of 20, 36, 52, and 64 min. To prepare for antibody incubation, the slide temperature is set to 36 °C and maintained for 4 min. The primary antibody application involves depositing one drop of CD68(KP-1), covering it with Liquid Covering Solution (LCS), and incubating for 32 min. A blocking step is then performed using the Ultrablock function, followed by reapplication of CD68, covering with LCS, and incubating for 8 min. The counterstaining process involves applying a drop of hematoxylin, covering with LCS, and incubating for 8 min. Finally, post-counterstaining is carried out by applying a drop of bluing reagent, covering with LCS, and incubating for 4 min.

## 3. Results

### 3.1. Patients

**Figure 1:**
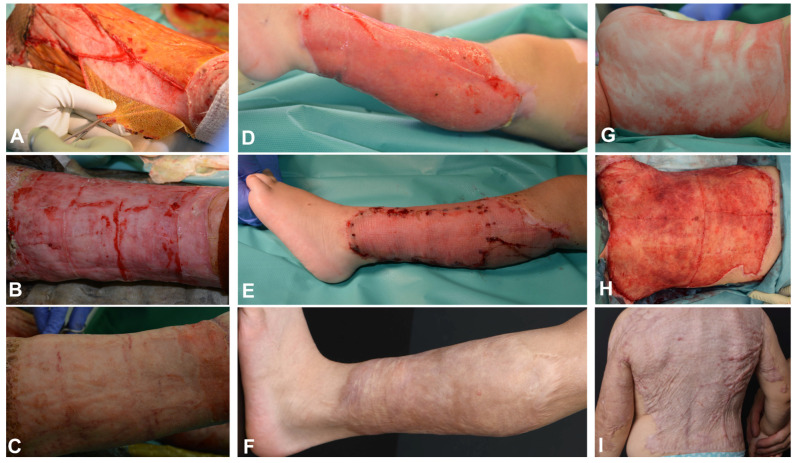
Wound progression of patients 1–3. (**A**–**C**) Patient 1: (**A**) Delamination of NovoSorb^®^ Biodegradable Temporizing Matrix (BTM) 31 days post-application shows a stable neodermis before the application of cultured dermal epidermal autografts (CDEAs); (**B**) 100% engraftment of CDEAs observed one week after transplantation of CDEAs on NovoSorb^®^ BTM; (**C**) stable skin condition observed three weeks post-transplantation of CDEAs on NovoSorb^®^ BTM. (**D**–**F**) Patient 2: (**D**) 21 days post-initial coverage with NovoSorb^®^ BTM, showing a fully vascularized neodermis; (**E**) five days post-split-thickness skin graft (STSG) on NovoSorb^®^ BTM application, demonstrating 100% graft take; (**F**) 1.5 years post-STSG on NovoSorb^®^ BTM transplantation, illustrating a flat, elastic, and fully integrated skin graft. (**G**–**I**) Patient 3: (**G**) Initial presentation showing full-thickness scald (oil) injury; (**H**) shortly before skin transplantation: delaminated NovoSorb^®^ BTM observed 28 days after its application; (**I**) one year after transplantation of MEEK micrografts, illustrating the skin graft is flat, elastic, and fully integrated.

**Figure 2:**
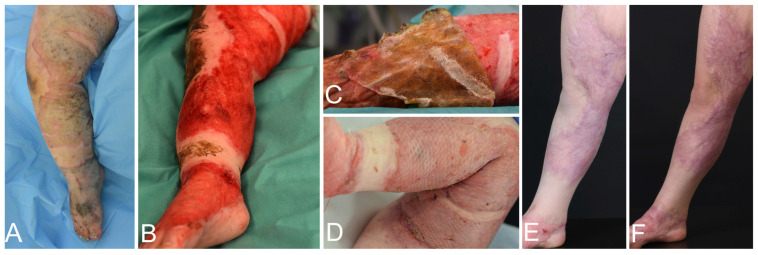
Wound progression of patient 4. (**A**) Initial presentation showing full-thickness scald injury. (**B**,**C**) Delamination of NovoSorb^®^ BTM 27 days post-application, showing a well-vascularized neodermis. (**D**) Depiction of 100% take of the meshed split-thickness skin graft (STSG) on NovoSorb^®^ BTM, observed 7 days post-transplantation. (**E,F**) Depiction of 2.5 and 4 years, respectively, post-transplantation of the STSG on NovoSorb^®^ BTM, illustrating the skin graft is flat, elastic, and fully integrated.

Between January 2020 and September 2022, four pediatric patients treated at the pediatric burn center of the University Children’s Hospital Zurich for full-thickness thermal injuries affecting 25–95% of their total body surface area (TBSA) were included in the study. For patient demographics and characteristics, treatment sequence, and grafting modalities of the thermal injury see Table 1; for photographic examples see Figure 1 and Figure 2. Every patient underwent standard treatment including surgical debridement, escharotomy if necessary, excision, wound bed conditioning using allografts, and silvercoated wound dressings before application of NovoSorb^®^ BTM on the full-thickness skin defects. After NovoSorb^®^ BTM integration and vascularization (27–41 days post-placement), the thin sealing membrane of the NovoSorb^®^ BTM was removed by delamination to enable the placement of the autograft as the second step. Various autologous grafting techniques were used, including STSG as sheet graft, and as MEEK micrografts, CEAs, CDEAs, and dS. Successful wound coverage was achieved with excellent take in all cases and grafts remained robust and stable throughout the study period. Multiple punch biopsies (1–2 per patient and time point) were taken at different time points after application of NovoSorb^®^ BTM and the respective autograft as seen in Table 1.

### 3.2. Epidermal Compartment and Dermo-Epidermal Junction

**Figure 3:**
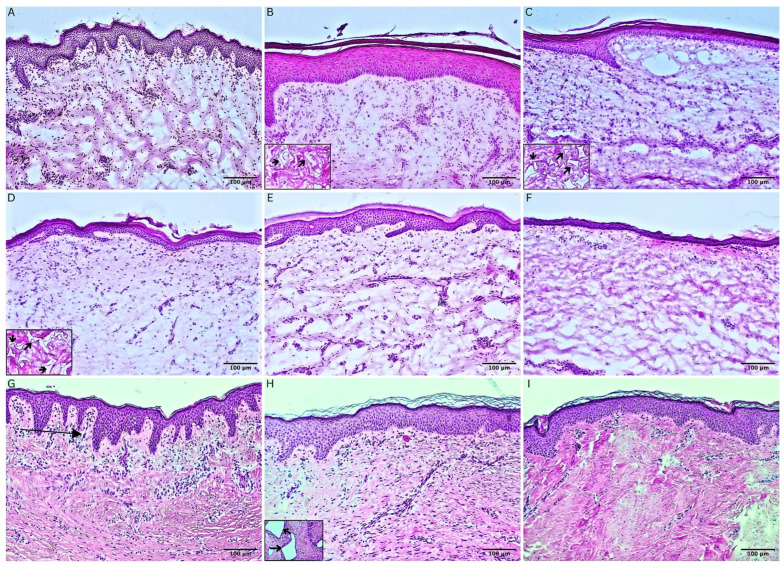
Haematoxylin and eosin (H&E) staining showing epidermal architecture with the formation of rete ridges, delineating the epidermal–dermal border, and the presence of NovoSorb^®^ Biodegradable Temporizing Matrix (BTM) remnants over time. Images depict normal skin and skin biopsies taken 1.5 months to 2.6 years after application of NovoSorb^®^ BTM in patients 1–4. (**A**) Normal human foreskin as control. (**B**–**F**) Patient 1: (**B**) 1.5 months post-application of NovoSorb^®^ BTM (covered with cultured dermo-epidermal autografts (CDEAs)), NovoSorb^®^ BTM material is visible as indicated by the black arrows in the magnification; (**C**) 2.5 months post-application of NovoSorb^®^ BTM (covered with denovoSkin™ (dS)), NovoSorb^®^ BTM material is visible as indicated by the black arrows in the magnification; (**D**) 3.5 months post-application of NovoSorb^®^ BTM (covered with dS), NovoSorb^®^ BTM material is visible as indicated by the black arrows in the magnification; (**E**) 5.5 months post-application of NovoSorb^®^ BTM (covered with dS), no NovoSorb^®^ BTM remnants visible with H&E staining; (**F**) 2.4 years post-application of NovoSorb^®^ BTM (covered with dS), no NovoSorb^®^ BTM remnants visible with H&E staining. (**G**) Patient 2: 8 months post-application of NovoSorb^®^ BTM (covered with split-thickness skin graft (STSG)). Black arrows indicate rete ridges; no NovoSorb^®^ BTM remnants visible. (**H**) Patient 3: 1.4 years post-application of NovoSorb^®^ BTM (covered with MEEK micrograft); no NovoSorb^®^ BTM remnants visible in H&E staining, but black arrows in the magnification indicate the original location of NovoSorb^®^ BTM. (**I**) Patient 4: 2.6 years post-application of NovoSorb^®^ BTM (covered with STSG), no NovoSorb^®^ BTM remnants visible in H&E staining. Scale bars: 100 µm.

To assess the epidermal compartment and the dermo-epidermal junction (DEJ), several stainings were performed and analyzed. As shown in the H&E staining of Figure 3, all skin biopsies ranging from 1.5 months up to 2.6 years after application of NovoSorb^®^ BTM (Figure 3B–I) and the respective autograft placed on top showed a multilayered, cornified epithelium almost indistinguishable from the epidermal architecture of normal skin (Figure 3A). However, the rete ridges were not as prominently developed in all biopsies as in normal skin. Depending on the autograft applied to cover the NovoSorb^®^ BTM, the degree of rete ridge formation differed. In biopsies with coverage of STSG as sheet grafts, rete ridges were pronounced and comparable to normal skin. In contrast, in skin biopsies after application of STSG as MEEKs and cultured skin products (CDEAs/CEAs and dS) on top of the NovoSorb^®^ BTM, rete ridges were less pronounced or flat at the time of the biopsy.

**Figure 4:**
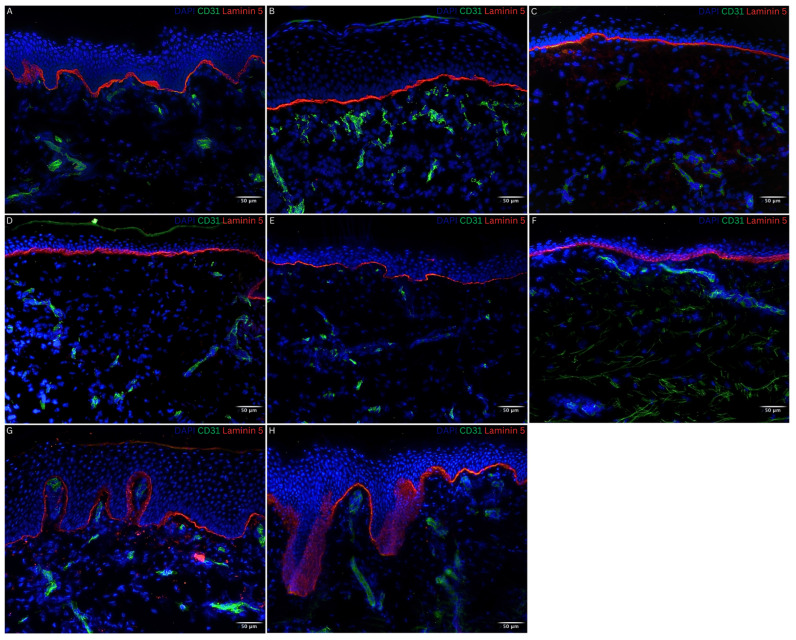
Visualization of the basement membrane (Laminin 5) and of dermal vascularization (CD 31) by immunofluorescence staining in normal skin and skin biopsies taken 1.5 months to 2.6 years post-application of NovoSorb^®^ Biodegradable Temporizing Matrix (BTM) from patients 1, 3, and 4. CD31 (green), Laminin 5 (red), DAPI (blue) staining cell nuclei. (**A**) Normal human foreskin as control. (**B**–**F**) Patient 1: (**B**) 1.5 months post-application of NovoSorb^®^ BTM (covered with CDEAs); (**C**–**E**) 2.5, 3.5, and 5.5 months post-application of NovoSorb^®^ BTM (covered with dS); (**F**) 2.4 years post-application of NovoSorb^®^ BTM (covered with CDEAs). (**G**) Patient 3: 1.4 years post-application of NovoSorb^®^ BTM (covered with MEEK micrografts). (**H**) Patient 4: 2.6 years post-application of NovoSorb^®^ BTM (covered with STSGs). The basement membrane was well developed and continuous across the entire interface between the epidermal and dermal layers in all patient biopsies. Consistent dermal vascularization was observed throughout all samples. Scale bar: 50 µm.

To evaluate the DEJ, an immunofluorescence staining of Laminin 5, a component of the basement membrane, was performed. The basement membrane was properly developed and uninterrupted throughout the entire contact area between the epidermal and dermal layer of all patient biopsies regardless of the nature of the autograft transplanted, reflecting the histological aspect of normal skin (Figure 4B–H).

### 3.3. Dermal Compartment

**Figure 5:**
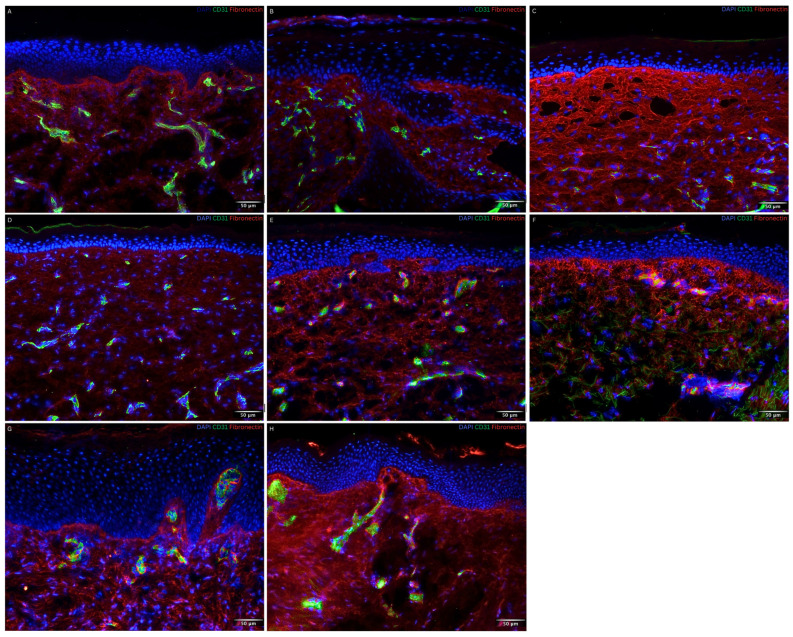
Visualization of the extracellular matrix (fibronectin) and of dermal vascularization (CD31) by immunofluorescence staining in normal skin and skin biopsies taken from 1.5 months to 2.6 years post-application of NovoSorb^®^ Biodegradable Temporizing Matrix (BTM) from patients 1, 3, and 4. Fibronectin (red), CD31 (green), DAPI (blue) staining cell nuclei. (**A**) Normal human foreskin as control. (**B**–**F**) Patient 1: (**B**) 1.5 months post-application of NovoSorb^®^ BTM (covered with cultured dermal epidermal autografts (CDEAs)); (**C**–**E**) 2.5, 3.5, and 5.5 months post-application of NovoSorb^®^ BTM (covered with denovoSkin (dS)); (**F**) 2.4 years post-application of NovoSorb^®^ BTM (covered with CDEAs). (**G**) Patient 3: 1.4 years post-application of NovoSorb^®^ BTM (covered with MEEK micrografts). (**H**) Patient 4: 2.6 years post-application (covered with STSGs). Migrated fibroblasts were evenly distributed within the scaffold structure. A prominent fibronectin stain was detected along the entire dermo-epidermal junction (DEJ) in all biopsies, comparable to that of normal skin, beginning at 1.5 months post-application of NovoSorb^®^ BTM. Consistent dermal vascularization was observed throughout all samples. Scale bar: 50 µm.

To show the distribution of the vascular network within the dermal compartment, an immunofluorescence staining for CD31 was performed. Endothelial cells reached up towards the dermo-epidermal layer, forming a vascular network (Figure 4 and Figure 5B–D). As seen in the timeline of Figure 4 and Figure 5**,** the vessels/capillaries remained evenly present in all biopsies up to 2.6 years post-application of NovoSorb^®^ BTM.

To demonstrate the production of extracellular matrix components by fibroblasts, a fibronectin immunofluorescence staining was performed. As seen in Figure 5, migrated fibroblasts were evenly distributed within the scaffold structure. Beneath the DEJ, along its entire length, a prominent fibronectin stain was detected in all biopsies comparable to normal skin. This was seen as of 1.5 months post-application of NovoSorb^®^ BTM (Figure 5B).

**Figure 6:**
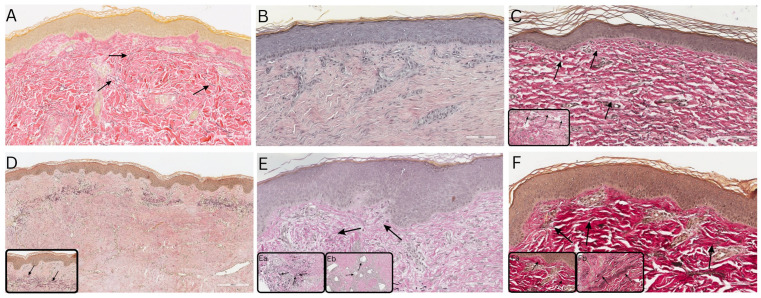
Elastica van Gieson (EvG) staining of collagen (red) and elastic (black) fibers in normal skin and skin biopsies taken 8 months to 2.6 years post-application of NovoSorb^®^ Biodegradable Temporizing Matrix (BTM), illustrating collagen and elastic fiber architecture over time in the newly formed dermal layer from patients 1–4. (**A**) Human foreskin as control, showing normal development of rete ridges. Collagen and elastic fibers are unevenly distributed throughout the dermis. Elastic fibers are indicated by black arrows. (**B**,**C**) Patient 1: (**B**) 1.5 years post-application of NovoSorb^®^ BTM (covered with cultured dermal epidermal autografts (CDEAs)), densely arranged collagen fibers show a predominantly homogenous arrangement compared to normal skin; no elastic fibers are visible; (**C**) 2.4 years post-application of NovoSorb^®^ BTM (covered with denovoSkin (dS)), collagen fibers show a tendency to uneven distribution, with elastic fibers integrated into the upper dermal layer (black arrows in magnification). (**D**) Patient 2: 8 months post-application of NovoSorb^®^ BTM (covered with split-thickness skin grafts (STSGs)), densely and rather homogenously arranged collagen fibers in the neodermis, with clusters of elastic fibers within the dermal remnant of the transplanted STSG (black arrows in magnification); prominent rete ridges are observed; (**E**) Patient 3: 1.4 years post-application of NovoSorb^®^ BTM (covered with MEEK micrografts), densely (with tendency to uneven distribution) arranged collagen fibers in the neodermis, clusters of elastic fibers (**Ea**) (black arrows in (**E**) and in (**Ea**) show elastic fibers) and gaps indicating areas where the NovoSorb^®^ BTM has dissolved (**Eb**, black arrows). (**F**) Patient 4: 2.6 years post-application of NovoSorb^®^ BTM (covered with STSGs) show rather disorganized collagen fiber distribution comparable to normal skin, with clusters of elastic fibers (black arrows in Figure **F** and magnifications **Fa** and **Fb**) and prominent rete ridges. Scale bar: 100 µm.

The EvG staining (Figure 6) further visualizes collagen and elastic fibers indicating the presence of a newly formed dermal layer at different time points in all patients. Normal skin, as a control, showed disorganized collagen fibers (Figure 6A). Collagen was observed throughout the entire dermis in all biopsies, indicating that the dermal scaffold provided by the NovoSorb^®^ BTM had been entirely replaced, which could be seen as soon as eight months after NovoSorb^®^ BTM application in our biopsies. In Figure 6B,C, two time points of the same patient are visualized. In Figure 6B, a relatively homogeneous alignment of collagen fibers is observed, in contrast to the disorganized pattern seen in healthy skin (Figure 6A). Conversely, Figure 6C displays a tendency towards a more disorganized arrangement, bearing greater similarity to the natural, irregular collagen structure illustrated in Figure 6A. A dynamic alteration in collagen fiber organization is evident between the two time points. The collagen fibers within the newly formed dermal layer, as depicted in Figure 6D,E, exhibit a pattern more closely aligned with the homogenous organization observed in Figure 6B. In contrast, the collagen arrangement in Figure 6F displays greater resemblance to the irregular, natural pattern characteristic of healthy skin, as shown in Figure 6A.

Regarding elastic fibers, various patterns could be seen within the patients’ samples. Elastic fibers were not visible one and a half years after NovoSorb^®^ BTM and autograft (CDEA) transplantation (Figure 6B), whereas they became apparent 2.4 years after NovoSorb^®^ BTM and autograft (dS) transplantation (Figure 6C). Figure 6D shows the appearance of elastic fibers only within the transplanted dermal remnant of STSG and no elastic fibers within the newly formed dermal layer eight months after application of NovoSorb^®^ BTM. Figure 6E showed only singular elastic fibers within the newly formed dermal layer and one area of densely congregated elastic fibers (Figure 6Ea). Similar to Figure 6C, in Figure 6F and the respective inserts Figure 6Fa,Fb, elastic fibers are visible in the newly formed dermal layer directly below the DEJ (likely remnants of dermal layer of STSG) as well as deeper towards the subcutis (newly formed) only at 2.6 years after NovoSorb^®^ BTM and autograft transplantation (STSG).

**Figure 7:**
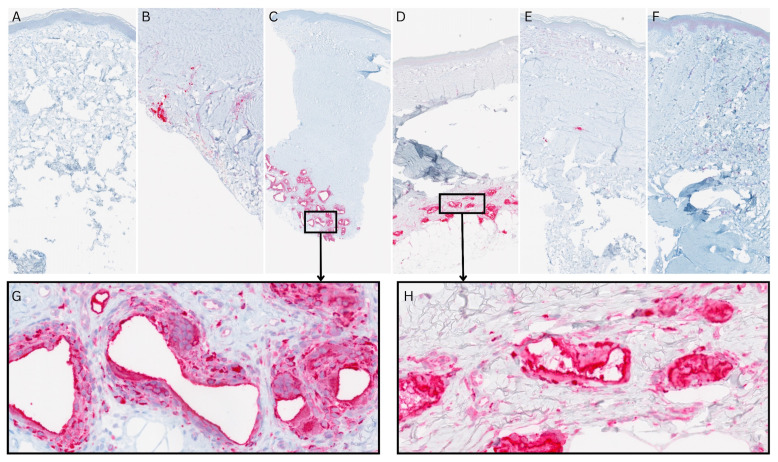
CD68 immunohistochemistry staining of macrophages, partly responsible for degrading. NovoSorb^®^ Biodegradable Temporizing Matrix (BTM) in skin biopsies taken from 8 months to 2.6 years post-application of NovoSorb^®^ BTM from patients 1–4. (**A**) Normal human foreskin as control, showing no visible macrophages. (**B**) Patient 2: At 8 months post-application of NovoSorb^®^ BTM (covered with split-thickness skin graft (STSGs)), few macrophages are visible throughout the neodermis. (**C**) Patient 3: At 1.4 years post-application of NovoSorb^®^ BTM (covered with the MEEK technique), macrophages cluster around areas where NovoSorb^®^ BTM has dissolved as shown in the magnification (**G**). (**D**,**E**) Patient 1: (**D**) 1.4 years post-application of NovoSorb^®^ BTM (covered with cultured dermal epidermal autografts (CDEAs)), macrophages cluster around areas where NovoSorb^®^ BTM has dissolved as shown in the magnification (**H**); (**E**) at 2.4 years post-application of NovoSorb^®^ BTM (covered with denovoSkin (dS)), few macrophages are seen within the neodermis or epidermal layer. (**F**) Patient 4: 2.6 years post-application of NovoSorb^®^ BTM (covered with STSGs), few macrophages are seen within the neodermis or epidermal layer.

A CD68 immunohistochemistry was performed to visualize macrophages, which are partly responsible for degrading NovoSorb^®^ BTM [27]. As shown in Figure 7B–D, a granulomatous reaction consistent with a foreign body response was observed. It could be seen as early as eight months post-application primarily at the interface of the transplanted NovoSorb^®^ BTM to the subcutaneous layer and persistent up to 1.4 years post-application.

The dermal compartment was examined for persistence of polyurethane NovoSorb^®^ BTM remnants, which are degraded by macrophages over time. As seen in the inserts of the H&E stainings in Figure 3B–D, particles of NovoSorb^®^ BTM could be identified up to 3.5 months after application. No more NovoSorb^®^ BTM remnants could be found in our biopsies at later time points. However, in Figure 3H the spaces remained, in which NovoSorb^®^ BTM used to reside, and which were not entirely filled with components of the extracellular matrix. Similarly, in Figure 6Ea gaps of the NovoSorb^®^ BTM remnants that were not filled up by the extracellular matrix were visible.

**Figure 8:**
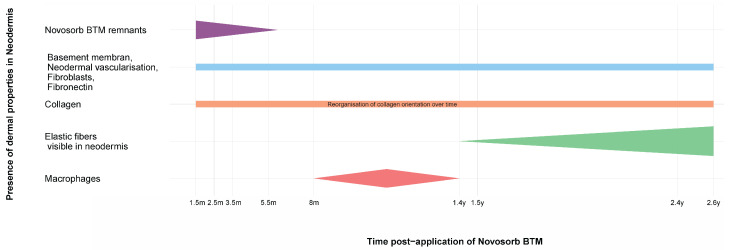
Timeline of biopsy results following NovoSorb^®^ BTM application.

Figure 8 shows a summary of the components of the neodermis and their developmental course over time. 

Progression of dermal properties in a newly formed neodermis following the application of NovoSorb^®^ BTM in patients 1–4 with full-thickness skin injuries, over a timeline spanning 1.5 months to 2.6 years post-application, is detailed below.

*Y-Axis (Dermal Properties in the Regenerated Dermis):*

- NovoSorb^®^ BTM remnants: Visible at early time points (up to 3.5 months) but completely resorbed by 5.5 months;
- Basement membrane, dermal vascularization, migrated fibroblasts, and fibronectin: The basement membrane becomes continuous across the dermo-epidermal junction (DEJ), with consistent vascularization observed at all time points. Migrated fibroblasts were evenly distributed within the scaffold structure, and fibronectin was prominently detected along the DEJ;
- Collagen fibers: First observed at 1.5 months, becoming increasingly visible up to 2.6 years with a dynamic change in organization pattern over time;
- Elastic fibers: Increasingly visible within the neodermis at 1.4 years to 2.6 years;
- Macrophages: Active in early phases around NovoSorb^®^ BTM remnants, with activity diminishing as the dermis matures.

*X-Axis (Timeline):* Represents biopsy collection points spanning from 1.5 months to 2.6 years after NovoSorb^®^ BTM application.

## 4. Discussion

In our study, we histologically observed the development of full-thickness thermal injuries in pediatric patients successfully treated with NovoSorb^®^ BTM and subsequently with various autografts up to 2.6 years after application. In general, we could see a gradual transformation of the dermal compartment with features comparable in architecture and composition to healthy skin. Several aspects hereof deserve a more detailed discussion.

### 4.1. Clinical Aspect

Clinically, we found a rapid integration of NovoSorb^®^ BTM as soon as 27 days post-application in all our patients. At removal of the sealing membrane, a macroscopically visible sufficiently vascularized newly formed dermal layer was present. Several previous publications have shown similar results with a matured NovoSorb^®^ BTM as soon as three weeks after application allowing for autografts to be transplanted in a second step [9,11,12,13,14,15,16,17,18,19]. Even though different kinds of autografts—non-cultured and cultured—were applied to the delaminated NovoSorb^®^ BTM in our patients, all grafts showed rapid adherence with development of a robust epidermal barrier. This is in alignment with results shown in the literature, confirming the ability of NovoSorb^®^ BTM to accommodate different methods of grafting [28].

### 4.2. Histological Analysis

Histologically, biopsies taken from the various patients at multiple time points up to 2.6 years after NovoSorb^®^ BTM application showed several different aspects. Independent of which type of autograft was used to cover NovoSorb^®^ BTM, a well-developed and stratified epidermis was seen in all biopsies at all time points. Hence, we conclude that NovoSorb^®^ BTM, or rather the developed neodermis based on NovoSorb^®^ BTM, respectively, enable the autografts and the containing keratinocytes to regularly renew its epidermal layer. Additionally, no indication of adverse effects on keratinocytes and the epidermal compartment was seen over the entire timespan. Equal results have been described in in vitro studies and in animal models [20,29], where NovoSorb^®^ BTM was seeded with human-derived keratinocytes, showing high survival rates of the keratinocytes and stability of its newly formed epidermis as well as attachment to the newly formed neodermis. Additionally, Schiestl et al. [22] and Greenwood et al. [21] showed equal results in clinical application on patients suffering from extensive full-thickness burns treated with NovoSorb^®^ BTM and various autografts in a two-step procedure.

Analyzing the dermo-epidermal area of our samples, considerable differences could be detected. When STSG sheet grafts were transplanted, rete ridges were prominent. With all other autografts applied, the interface between the dermis and the epidermis remained flat, even after several years. Staining of Laminin 5, highlighting a component of the basement membrane, nonetheless showed a continuous signal throughout the entire length of the dermo-epidermal contact area of all samples, indicating the formation of a normal, adhering, and functioning dermo-epidermal junction and strong connection between the two compartments. What must be mentioned is the fact that some cultured autografts used, namely CDEAs and, in particular, dS, contain their own dermal component, thereby adding a certain dermal layer in between the NovoSorb^®^ BTM-based neodermis and the epidermal compartment, diminishing direct contact with the NovoSorb^®^ BTM-based neodermis. But, regardless of which graft was used to cover the vascularized, fully integrated, and matured NovoSorb^®^ BTM in our patients, connection with and development of the underlying dermal layer was uniformly possible and allowed for a strong interaction of the two compartments. This is an important factor, as the DEJ plays a crucial role in enabling skin homeostasis, function, and stability and is a key feature of skin integrity and communication between the epidermal and dermal layers [30].

Several components of the dermal compartment were analyzed to observe the development and reorganization of a neodermis based on the dermal substitute NovoSorb^®^ BTM. The vascular network, illustrated by stained endothelial cells, was present as of the first time points of biopsies taken, reaching from the subcutis, throughout the newly formed dermal layer, up to the dermo-epidermal junction. Blood vessels continued to remain evenly present in all biopsies up to 2.6 years post-application of NovoSorb^®^ BTM, which is an important hallmark of homeostasis. The only study regarding this topic reported on in the literature was made on male athymic nude mice treated with NovoSorb^®^ BTM and human keratinocytes [5,20]. They showed a different vessel density at diverse stages of wound repair with decreased vessel density after six months compared to the initial vessel density seen, which was explained as achieved homeostasis at this time point. This seems to be due to the large pores in the NovoSorb^®^ BTM matrix and lack of crosslinks unlike Integra^®^ Dermal Regeneration Template, which facilitate the infiltration of cellular materials and significantly enhance the vascularization of the newly formed neodermis [20]. The time intervals and locations of the biopsies taken in this study varied, making it impossible to perform a dynamic analysis of the blood vessel influx into the matrix and confirm this finding. However, the appearance of the vascular network seemed stable, suggesting that a homeostatic state was always achieved for all patients.

Another important cellular dermal feature are fibroblasts, ensuring synthesis and deposition of extracellular matrix components rendering the dermal component its important characteristic features such as strength and elasticity. As an indirect marker for fibroblasts, its products fibronectin, collagen, and elastic fibers, three of the main components of the extracellular matrix, were stained [31]. In our samples, these fibroblast-produced extracellular matrix components were visible as of 1.5 months post-application of NovoSorb^®^ BTM, except for elastic fibers, which required more time for production. As these extracellular components are not present in native NovoSorb^®^ BTM, fibroblasts attracted by and grown into the matrix must have deposited them. Over time, the regeneration process of the neodermis showed a dense fibronectin and collagen construct intertwined with the newly formed vascular network throughout the entire dermis up to the DEJ (Figure 4 and Figure 5). This indicates that NovoSorb^®^ BTM provides an appropriate environment for fibroblasts and vessels to be attracted into its matrix, to stimulate growth, cell multiplication, and physiological function demonstrated by the formation and deposition of ECM components. All cellular and acellular components depicted in our analysis make up for the “neodermis” formed based on the presence of a dermal template, a persisting and self-renewing newly formed dermal compartment functioning within itself and as a support for various autografts applied on top [32].

The collagen fibers observed in our experimental samples exhibited a distinctly uniform alignment up to 1.5 years post-BTM application, contrasting with the randomized orientation characteristic of healthy control tissue. This pattern closely resembles the organized collagen structure seen in scar tissue formation [33], suggesting that a similar process of fibrotic organization might be occurring in our samples initially. While components characteristic of healthy skin were present, the collagen fiber organization diverged from the complex, multidirectional pattern observed in non-scarred dermis, reinforcing the distinction between regenerating and scar tissue morphologies. However, changes in collagen fiber arrangement are evident at the 2.4- and 2.6-year post-NovoSorb^®^ BTM application time points, suggesting a dynamic process and a development towards a normal, uninjured skin pattern rather than a rigid, hypertrophic, and fibrotic scar formation. An animal model study by Stefanelli et al. demonstrated that the porous structure of BTM facilitated the parallel alignment of collagen fibrils during dermal regeneration. Notably, this organized collagen deposition did not result in any observable increase in wound contracture [34]. In a porcine model by Greenwood et al., predominantly collagen type 1 was observed as thick dense bundles by day 112 post-application of BTM but loosely organized [35]. The long-term patient study by Greenwood et al. demonstrated, through Masson’s trichrome staining, that collagen bundles in the papillary and reticular dermal layers appeared thick, loose, and irregular in uninjured skin. In contrast, the treated areas exhibited thin, densely packed collagen fibers with parallel alignment, indicative of dermal fibrosis at day 528 post-application [21]. The findings collectively suggest that while NovoSorb^®^ BTM facilitates organized collagen deposition during the early phases of dermal regeneration, there is a gradual remodeling process over time that trends towards a more natural, multidirectional collagen arrangement characteristic of uninjured skin. This progression underscores the dynamic nature of collagen reorganization following BTM application, highlighting its potential to support not just scar mitigation but also long-term tissue normalization. Further longitudinal studies would be valuable to elucidate the extent to which this remodeling continues beyond the observed time points.

Elastic fibers, also produced by fibroblasts, were deposited in a slower and scarce manner within the newly formed neodermis compared to fibronectin and collagen. Although fibroblasts were present, elastic fiber deposition within the NovoSorb^®^ BTM-based neodermis was seen only as of 2 years after NovoSorb^®^ BTM application. Deposition also depended on autograft application. For instance, in the dermal component of dS application, elastic fibers were seen more in abundance than after other laboratory-grown autograft application. If, in our study, elastic fibers were visible earlier, as of eight months post-application of NovoSorb^®^ BTM, they were only visible within the thin dermal layer of the transplanted STSG (and therefore probably preexisting from the dermal component of the STSG) and not within the newly formed template-based dermal layer. In uninjured skin, elastic fibers within the papillary layer of the dermis are thin and perpendicular to the thicker elastic fibers within the reticular layer [36]. In contrast, injured skin displays an abnormal pattern of elastic fibers [36]. Our findings align with research on injured skin treated with Integra^®^ Dermal Regeneration Template and MatriDerm^®^—other dermal templates used for deep wound care—where few to no elastic fibers were visible in the neodermis initially, and those that were present displayed an abnormal pattern compared to probes of healthy skin [37]. In our study, elastic fibers became evident within the neodermis as of two years after NovoSorb^®^ BTM application, with a clear difference regarding which autograft was applied on top as preexisting elastic fibers would be co-transplanted with STSG as the autograft; however, definitive conclusions regarding their organizational pattern within the neodermis cannot be reliably drawn from our observations. The progression of elastic fiber appearance seen correlates with clinical observations of our patients, indicating an initial phase of rigidity in the healing skin, followed by a development of a more elastic scar over time.

To investigate the body’s immunological response to the presence of a foreign material such as NovoSorb^®^ BTM, macrophages were highlighted. Stainings showed only mild granulomatous reaction at the lower edges of NovoSorb^®^ BTM application and were visible even over one year post-application of NovoSorb^®^ BTM and the respective autograft. This seems surprising as no NovoSorb^®^ BTM remnants in our study were visible between the time points 3.5–5.5 months post-application of NovoSorb^®^ BTM. In studies conducted on animals treated with NovoSorb^®^ BTM, immune cells were observed, indicating an inflammatory and granulomatous response to the polymer comparable to that elicited by clinically established materials, such as sutures and the dermal regeneration template Integra^®^ [34,36]. The persistence of macrophages, even one year after application, may be attributed to their role in phagocytosing tissue debris and managing the controlled degradation of NovoSorb^®^ BTM. The material’s gradual biodegradation requires sustained macrophage activity to clear breakdown products and support the remodeling process, thereby accounting for the higher macrophage presence over an extended period [34]. Similar results were observed in human biopsies, where patients treated with NovoSorb^®^ BTM displayed macrophages without evidence of excessive or irregular immune responses [28]. A short-term (21 days) in vitro study by Banakh et al. [20] showed NovoSorb^®^ BTM grafts induce a stronger and prolonged inflammatory response compared to Integra^®^, with elevated levels of cytokines such as IL-6 and TNF-α, which promote extended proliferation and vascularization. This contrasts with the earlier transition to ECM remodeling seen in Integra^®^, driven by its collagen content. Although macrophages were referenced in this study, a direct and distinct staining to differentiate M1 and M2 macrophage subtypes was not performed. Consequently, their conclusions regarding macrophage polarization remain indirect, relying solely on the inflammatory markers assessed rather than definitive immunohistochemical evidence: macrophages play a pivotal role in this process, with BTM likely involving higher M1 macrophage activity, while Integra^®^ facilitates a shift towards M2 macrophages, supporting inflammation resolution. M1 macrophages are associated with the production of pro-inflammatory cytokines, such as IL-1, IL-6, TNF-α, and IFN-γ, which drive the inflammatory and early proliferative phases of wound healing. In contrast, M2 macrophages, stimulated by IL-4 and IL-13, are linked to anti-inflammatory and reparative functions, producing cytokines like IL-10 and TGF-β that promote ECM remodeling and tissue repair. The balance of M1/M2 polarization and the IL-10/IL-6 ratio further influences the distinct healing outcomes observed in these grafts [20].

Wound healing with NovoSorb^®^ BTM is marked by a robust inflammatory response that promotes rapid neodermis formation and vascularization. These processes create a supportive environment for skin grafting and help minimize scarring. Compared to biological matrices such as Integra^®^, NovoSorb^®^ BTM generates higher levels of pro-inflammatory cytokines. However, its porous structure plays a pivotal role in attenuating mechanical signals that drive fibroblast-to-myofibroblast conversion, effectively limiting wound contraction and fibrosis as described by Rajaram et al. [38]. Hence, as observed earlier, all important features of near-normal skin architecture were seen in all patients suggesting no irregularities or immune response reactions provoked by NovoSorb^®^ BTM application that could indicate a rejection reaction.

A further interesting finding is the presence of NovoSorb^®^ BTM remnants. In our samples, we could detect remaining NovoSorb^®^ BTM particles mainly within the basal layers of the neodermis until 3.5 months post-application of NovoSorb^®^ BTM. This correlates with early investigations of NovoSorb^®^ BTM in animal models, which describe degradation rates between 3–6 months post-application of NovoSorb^®^ BTM [39]. As clinically observed by Wagstaff et al., the persistence of degradation processes up to 18 months post-application may account for the residual gaps of NovoSorb^®^ BTM observed in patient 3, even at 1.4 years following its application [28].

Studies showing human histological features of skin defects treated with NovoSorb^®^ BTM are scarce, as visualized in Table 2. Greenwood et al. [21], Schiestl et al. [22], Wagstaff et al. [28], and to a very limited extend Meuli et al. [4] showed long-term clinical success in the treatment of extensive full-thickness defects treated with NovoSorb^®^ BTM and various autografts with histologically confirmed similarity of the neodermis and epidermis to normal skin in a single patient each. In our histological study samples of four different pediatric patients analyzed over a long period of time, we showed developmental steps of dermal regeneration and neoformation up to 2.6 years after application. Different key components of dermal architecture reflecting various dermal features that eventually aid important functions and improvement of the patients’ quality of life could be visualized and showed features of a near-normal skin architecture such as a cornified, multilayered epidermis, a continuous DEJ, a fibronectin- and collagen-dense neodermis with a vast vascular system, and gradual elastic fiber formation. These are all features that have, to our knowledge, not been shown to this extent so far. Our results also lead us to the definition of the “neodermis”, a term often used in various situations and never in a standardized manner. We conclude that a “neodermis” is a dermal compartment featuring all cellular and acellular components developed by the patient themself, facilitated by using a dermal template.

Nonetheless, some limitations of this project exist. Although spanning a long period of time, the sample size presented here is small. Additionally, biopsies were taken of different body areas and of children only. Ideally, a prospective study with larger patient numbers and different age groups should be conducted to enable a comparison of areas treated with and without NovoSorb^®^ BTM or with other dermal templates applied so as to see if age plays a role in the regeneration process. This could offer further insights regarding clinical relevance in the use of dermal templates for treating severely burned patients and large full-thickness skin defects.

## 5. Conclusions

This is, to the best of our knowledge, the first long-term histological analysis and, in particular, the first analysis of severely burned pediatric patients treated with NovoSorb^®^ BTM in a two-step procedure. Regardless of which method was used to cover the developed NovoSorb^®^ BTM-based neodermis, a close-to-normal skin architecture could be detected. The results enhance our comprehension of dermal template function, development, and integration and lead us to define the term “neodermis”. NovoSorb^®^ BTM facilitates the gradual deposition of extracellular matrix components, such as collagen and elastic fibers, by fibroblasts. Initially, these fibers may exhibit an organizational pattern characteristic of inelastic scar tissue; however, over time, they increasingly approximate the structure seen in uninjured skin. This progressive remodeling suggests potential for achieving elastic scar formation, which may ultimately reduce functional impairments associated with inelastic scarring.

This seems to be a useful method rendering burn surgeons an additional tool to not only enable simple coverage of large full-thickness skin defects but also to aim for the best possible patient long-term outcomes with improvement of skin aesthetics, elasticity, and overall function.

## 6. Outlook

Given the small sample size in this study, further research is warranted with a prospective study and larger cohort spanning a longer duration, encompassing both pediatric and adult populations, to improve our understanding of neodermal regeneration. Additionally, comparative analyses with other established dermal regenerative templates, such as the Integra^®^ Dermal Regeneration Template and MatriDerm^®^, would be valuable to better elucidate the relative benefits of each template in facilitating neodermal development.

## Figures and Tables

**Table 1:** Data of the pediatric patients with full-thickness thermal injuries and time points of NovoSorb^®^ Biodegradable Temporizing Matrix (BTM) application with corresponding autograft transplantation.

Patients	1	2	3	4
Age at thermal injury	14 yrs	1.4 yrs	2 yrs	1.3 yrs
Gender	Male	Female	Female	Female
Cause of thermal injury	Flame injury	Flame injury	Scald injury	Scald injury
TBSA affected (%)	95	37	37	25
Grade of thermal injury	3	2–3	2–3	3
Time between step 1 and 2 (days)	31–41	37	28	27
Autograft	CEAs/CDEAs/dS	STSGs	MEEK	STSG
Time of biopsy after BTM application	1.5 m–2.4 yrs	8 m	1.4 yrs	2.6 yrs

yrs = year; m = month; TBSA = total body surface area; STSGs = split-thickness skin grafts; CEAs/CDEAs = cultured epidermal autografts/cultured dermo-epidermal autografts; dS = denovoSkin™, a complex cultured dermo-epidermal skin substitute.

**Table 2:** Summary of relevant studies with histological analysis of NovoSorb^®^ BTM. The table summarizes studies specifically focused on NovoSorb^®^ BTM, categorized by their methodology and focus. Columns indicate whether the study was animal-based, human-based, and whether it was classified as preclinical or clinical. Only papers with histological analysis were included.

Paper	Preclinical	Animal-Based	Human-Based	Case Report	Histological Analysis
Schiestl et al. (2024) (“Long-term outcomes of a cultured autologous dermo-epidermal skin substitute in children: 5 year results of a phase I clinical trial”) [3]			X	X	X
Meuli et al. (2019) (“A Cultured Autologous Dermo-epidermal Skin Substitute for Full-Thickness Skin Defects: A Phase I, Open, Prospective Clinical Trial in Children”) [4]			X	X	X
Cheshire et al. (2016) (“Artificial dermal templates: A comparative study of NovoSorb^TM^ Biodegradable Temporising Matrix (BTM) and Integra^®^ Dermal Regeneration Template (DRT)”) [5]	X	X			X
Wagstaff et al. (2015) (“Free Flap Donor Site Reconstruction: A Prospective Case Series Using an Optimized Polyurethane Biodegradable Temporizing Matrix”) [12]			X	X	X
Banakh et al. (2020) (“A Comparative Study of Engineered Dermal Templates for Skin Wound Repair in a Mouse Model”) [20]	X	X			X
Dearman and Greenwood (2022) (“Long-term follow-up of a major burn treated using composite cultured skin”) [21]	X	X			X
Schiestl et al. (2021) (“Expanding into the future: Combining a novel dermal template with distinct variants of autologous cultured skin substitutes in massive burns”) [22]			X	X	X
Greenwood et al. (“Evaluation of NovoSorb™ novel biodegradable polymer for the generation of a dermal matrix Part 2: In-vivo Studies”) [29]	X	X			X
Stefanelli et al. (2023) (“Design matters: A comparison of natural versus synthetic skin substitutes across benchtop and porcine wound healing metrics: An experimental study”) [34]	X	X			X
Dearman et al. (2023) (“Comparison of biopolymer scaffolds for the fabrication of skin substitutes in a porcine wound model”) [35]	X	X			X

## Data Availability

The data presented in this study are available on request from the corresponding authors due to privacy and ethical restriction.
